# Adolescent and caregiver attitudes towards telemedicine use in pediatric nephrology

**DOI:** 10.1186/s12913-021-06506-0

**Published:** 2021-06-01

**Authors:** Yi Qiu, Sherry Coulson, Christopher William McIntyre, Brooke Wile, Guido Filler

**Affiliations:** 1 Medical Sciences, Schulich School of Medicine and Dentistry, University of Western Ontario, 1151 Richmond Street, N6A 3K7 London, ON Canada; 2 Children’s Health Research Institute, 345 Westminster Ave, N6C 4V3 London, ON Canada; 3 Department of Paediatrics, University of Western Ontario, 800 Commissioners Road East, E3-206, ON N6A 5W9 London, Canada; 4 Department of Medicine, University of Western Ontario, 800 Commissioners Road East,E3-206, ON N6A 5W9 London, Canada; 5 Department of Biophysics, University of Western Ontario, 800 Commissioners Road East, E3-206, Ontario N6A 5W9 London, Canada; 6 Department of Pathology & Laboratory Medicine, University of Western Ontario, 800 Commissioners Road East, E3-206, ON N6A 5W9 London, Canada

**Keywords:** Caregiver attitudes, Adolescent attitudes, Telemedicine, Clinic visits, Travel distance

## Abstract

**Background:**

Telemedicine is increasingly utilized as an alternative to in person consultation. Current pandemic conditions are providing additional impetus to virtual care delivery. We compared both adolescent and caregiver (parent or guardian) attitudes towards telemedicine (here as tertiary center to remote health care location) as a crucial determinant of longer-term effectiveness.

**Methods:**

This qualitative research study analyzed transcribed structured telephone interviews with both 11–18 year-old pediatric nephrology patients and their caregivers and performed a quantitative analysis of patient demographics, disease factors and distance to tertiary center vs. telemedicine center.

**Results:**

The study was conducted in a medium-sized tertiary pediatric nephrology centre with a large catchment area of over 0.5 million square kilometers and 629,000 children and adolescents under 18 years of age. Eleven dyads of adolescents and caregivers were enrolled. Five adolescents were male. The mean age of the adolescents was 14.4 ± 2.5 years (range 11.2–18.0). The median distance to our tertiary center was 191 km (range 110–1378 km). Four adolescents lived more than 500 km from our tertiary center. The 11 adolescents had a total of 334 in person visits (mean 30 ± 25) and 86 telemedicine visits (mean 8 ± 7). A ratio of 2:1 telemedicine to in-person visits was favored; with caregivers more in favor of remote care than adolescents. Qualitative analysis found that experiences with telemedicine were distinguished by *consultation-specific factors* and *contextual factors*. *Contextual factors* (travel/cost savings) were valued for telemedicine by adolescents and caregivers. *Consultation-specific factors*, such as the ability to show the doctor physical symptoms, were more valued during in-person consultations, especially by adolescents. The overall visit type preference was related to the nature of the consultation. For regular check-ups, and for adolescents with less complex needs, participants felt that telemedicine offered a comparable experience to in-person visits. Adolescents with more complex conditions preferred in-person visits.

**Conclusions:**

Indiscriminate transfer to chronic care predicated on mainly telemedicine approach is not compatible with user expressed attitudes (especially among adolescents). Accurately mapping models of care to these attitudes is an essential determinant of effective management and longer-term engagement with potentially life-long health challenges.

**Supplementary Information:**

The online version contains supplementary material available at 10.1186/s12913-021-06506-0.

## Key Points

**What is known about this subject?** Information around attitudes towards telemedicine is currently limited, and exclusively focused upon the caregiver. There are no systematic evaluations of the pediatric adolescent perspective available at all.

**What this study adds**: Adolescent’s attitudes towards telemedicine differ from that of their caregivers. In general, adolescents valued in-person visits. Technical issues were listed as the major disadvantages. This study supports a blended approach to ongoing care, rather than exclusively relying on remote interaction.

## Background

Telemedicine is increasingly used to provide clinical services to remote patients outside of tertiary care centers [1]. Telemedicine is advantageous in helping the provision of care to patients with the lack of mobility, decreased funding, and lack of staff situations faced by families in rural settings [2]. Specifically, significant challenges exist for pediatric patients living in remote locations who require attention from specialized physicians, [3]. given that outcomes are partially determined by distance from specialized centers [4]. Current pandemic conditions are further fueling adoption of virtual care delivery. Effective care is crucial to producing best care for patients but also to ensure that they remain engaged with health care and not lost in the transition to lifelong adult services.

Care in Ontario is widely dispersed. Ontario is twice the size of California but with only approximately one-third of the population; served by only 4 pediatric nephrology centers. Remote patients may live more than 1,400 km away from the tertiary center provided at The University of Western Ontario, London Health Science Centre. Telemedicine offers a means to provide timely care to patients in remote areas [5, 6]. Over a five year period 652,337 Ontario telemedicine network visits were recorded [6]. Recently, there has been a surge of virtual care due to the COVID-19 pandemic [7]. Information around attitudes towards telemedicine is limited, and exclusively focused upon the caregiver [8]. There are no systematic evaluations of the adolescent perspective available in the setting of pediatric nephrology.

With this qualitative research study and descriptive statistics, we aimed to explore the attitudes of adolescent chronic pediatric nephrology patients and their caregivers towards telemedicine visits in comparison to in-person visits to our tertiary center. We also asked participants to establish their position on how in-person visits should take place (in relationship to telemedicine visits) and studied the relationships of these preferences to relevant demographic and geographic factors. We hypothesized that the adolescents may have different attitudes towards telemedicine than their caregivers.

## Methods

### Brief Description of Virtual Encounters using the Ontario Telemedicine Network

The Ontario Telemedicine Network is one of the largest telemedicine networks in the world. It uses secure, encrypted two-way videoconferencing to provide access to care for patients in every hospital and hundreds of other health care locations across the province, where there typically is a nurse or a coordinator at the remote site. Physicians often use their desktop computers. During the COVID-19 pandemic, the network services were expanded to include handheld devices, laptops and other devices directly in the home of the patients. For the purpose of this study, we are focusing only on on-site delivery of telemedicine care in remote locations. Furthermore, we refer to London as to the tertiary care center.

### Study Design, Study Period, Population and Researchers

This was a study using demographic data, adolescent characteristics and structured telephone interviews with 15 standardized questions to pediatric nephrology patients aged 11–18 years of age and their caregiver. The study was approved local Research Ethics Board (file number 108,400). Written informed signed consent/assent was obtained for all adolescents from caregivers and written informed assent was obtained from consenting minors. Study dyads were approached in writing and enrolled between December 2017 and December 2019. The main inclusion criteria was having at least one telemedicine encounter. Twenty-four study invitation letters were sent out in concert with the usual reminder for clinic attendance. Eleven dyads were recruited. We used purposeful sampling [8] to recruit adolescents representative of our wide geographical catchment area. This qualitative sampling strategy allowed us to explore similarities and differences in visit preferences based on geographic location. The interview questions are provided in Additional file 1. Interviews were conducted by volunteer health sciences students (YQ and BW), who had no relationship with the dyads. Their occupation and their desire to conduct research was disclosed in the study invitation letter. The interviews were conducted from the London Health Science Centre Hospital.

### Quantitative Methods

Adolescent patient’s electronic medical record provided their address, demographics, visit number, diagnosis, disease severity by chronic kidney disease (CKD) stage, distance to our tertiary center, distance to telemedicine center (stratified by 500 km distance) and frequency of visits. We used simple descriptive statistics to analyze the data as appropriate. Continuous variables were analyzed visually for normal to determine the use of parametric or non-parametric tests [9]. Data were compared based on the distribution with either the t-test or the Mann-Whitney test accordingly. Categorical data were analyzed using Fisher’s exact test. Data were analyzed using GraphPad Prism version 5.0f for Mac, GraphPad Software, San Diego, CA, USA.

### Qualitative Methods

Structured telephone interviews were conducted, audio recorded and then transcribed using NoNotes (v.19.11.0) for iPhone and analyzed using NVivo 12 Mac (QSR International Pty Ltd, Melbourne, Australia). We held individual/confidential interviews over the phone with adolescents and caregivers; to minimize bias from the other participant’s opinions and views. To ensure participant privacy/confidentiality, caregivers and adolescents were instructed to answer the interview questions separately without influencing each other’s responses. To ensure adolescents were not monitored by the caregivers, adolescents with their own phones answered questions physically distant from caregivers. For adolescents without their own phones, caregivers were instructed to give the adolescents privacy. The interviews took a mean of 15 min. We examined the dyads to look for similarities and differences between the family members. Using comparative analysis, we stratified participants’ answers by travelling shorter distances as longer or shorter than 500 km.

The strategies of immersion and crystallization were used in order to identify key themes [10]. We used content analysis with an inductive process of comparative coding [11]. Open coding followed by axial coding allowed us to discover common categories of factors relating to preference of type of visit. Two researchers (YQ and SC) independently analyzed each interview, then met to discuss the emerging themes and categories. Where differences in coding arose, we reached consensus through a discussion of our interpretations with all co-investigators [12]. The recruitment of new participants was terminated as the identified themes reached saturation. While the study was planned for 40 dyads, we reached saturation after 11 dyads. Transcripts were not returned to participants.

## Results

### Patient Demographics

We enrolled 11 adolescent/caregiver dyads. Five adolescents were male. The mean age of the adolescents was 14.4 ± 2.5 years (range 11.2–18.0). The median distance to our tertiary center was 191 km (range 110–1378 km). The median traveling distance saved by using telemedicine was 190 km (range 88-1377 km) one way. Four adolescents lived more than 500 km from the tertiary center. The dyads had a total of 334 in person visits (mean 30 ± 25) and 86 telemedicine visits (mean 8 ± 7). The maximum of in person visits was 85 and the maximum of telemedicine visits was 21. There were 7 adolescents who had congenital conditions affecting them throughout life. One adolescent had received a kidney transplant, two had congenital genetic conditions, two had congenital anomalies of the kidneys and urinary tract, two had advanced CKD, one each had renovascular hypertension, diabetic nephropathy and nephrotic syndrome, respectively. Dyad demographic and geographical data are provided in Table 1, with some data only for the patients but not the caregivers.

**Table 1:** Dyad demographics and geographical information (caregiver age not known). Of note, the differences of travel times were perceived, all dyads shared the same home address

	Minimum	25 % Percentile	Median	75 % Percentile	Maximum	Mean	Std. Deviation
Adolescent Age [years]	11.2	-	-	-	18	14.42	2.545
# telemedicine Visits	1	-	-	-	21	7.8	6.8
# In person Visits	2	-	-	-	85	30.4	25.3
Distance center telemedicine [km]	110	172.3	191	542.8	1378	-	-
Distance Home London [km]	100	154.8	191	553.3	1378	-	-
Distance Home telemedicine [km]	1	1	1	11.5	54	-	-
Distance saved [km]	88	144	190	535.8	1377	-	-
Perceived Time to London (Caregiver) [min]	60	86.25	120	195	720	-	-
Perceived Time to center (Adolescent) [min]	60	87.5	120	120	330	-	-
Perceived Time to telemedicine (Caregiver)	5	-	-	-	37.5	14.8	11.0
Perceived Time to telemedicine (Adolescent) [min]	5	-	-	-	30	15.8	9.6
Preferred Ratio (Caregiver)	1	-	-	-	5	2.9	1.5
Preferred Ratio (Adolescent)	0	-	-	-	3	1.5	1.1

*km* kilometer, *min* minutes

Among the 11 adolescents, 5 (45 %) preferred telemedicine visits and 6 (55 %) preferred in-person visits. Of the adolescents preferring telemedicine, 2/5 live more than 500 km away from the tertiary center. Among the adolescents favoring visits to our tertiary care center, 4/6 patients live closer than 500 km away. This suggests that adolescents slightly preferred in-person visits regardless of distance to tertiary care centers. By contrast, only two caregivers preferred in-person visits whereas 3 preferred telemedicine, and the majority of 6 preferred a mixture. There was a significant difference between the adolescent and caregiver preferences (*p* = 0.0239, Fisher’s exact test).

The preferred ratio for care between telemedicine to in-person was a median of 3:1 (range 0–5) whereas the caregivers suggested a median of 1:1 (range 0–10, *p* = 0.1311, Mann Whitney test). When comparing distance and severity of the adolescent diagnosis, there was neither a significant difference by distance nor disease severity. The combined preferred ratio of telemedicine to in-person visits by all participants was 2:1. The results are summarized in Table 2.

**Table 2:** Dyad responses to preferred ratio (telemedicine to in-person) and most favored visit preference

Dyad #	Preferred ratio (adolescent)	Preferred ratio (caregiver)	Most favored visit (adolescent)	Most favored visit (caregiver)
1	2:1	1:1	telemedicine	telemedicine
2	3:2	3:1	In-person	-
3	2:1	1:1	telemedicine	telemedicine
4	-	-	In-person	50/50
5	0:1	0:1	In-person	London in-person
6	3:1	1:1	telemedicine	Depends on appointment
7	-	1:1	In-person	Depends on season
8	4:1	1:1	telemedicine	Depends on season
9	3:1	3:1	telemedicine	50/50
10	3:2	2:1	In-person	London in-person
11	5:1	10:1	In-person	telemedicine

### Qualitative Analysis

The initial 11 dyad interviews (representing interviews of 22 participants) were transcribed collectively but analyzed in a sequential order. We noticed saturation of themes after this initial group of 11 dyads, and therefore further recruitment was stopped. As thematic coding was performed in sequential order, we noticed that starting at around dyad 8, we were not adding any new coding groups, and were only grouping the codes into existing categories. This is how we determined that saturation of themes was reached. We identified a series of main themes reflecting the factors influencing adolescents’ and their caregivers’ preferences. Each of these themes is described in more detail below and summarized in Table 3. Illustrative quotes are presented as exemplars of participant responses.

Factors associated with preferences emerged into two categories: consult-specific factors or context-specific factors relating to participants preferences. Consult-specific factors were identified as those that occur within the clinical encounter itself. Context-specific factors, on the other hand, were factors that occurred outside of the medical visit itself. These factors were further categorized as advantages and disadvantages to telemedicine and in-person visits. In addition to these factors, we identified factors mitigating preference for one type of consultation over the other. Overall, we found that consult-specific advantages came out as the primary reasons for participants’ preference for in-person visits, while context-specific advantages were reasons for preferring telemedicine visits.

**Table 3:** Themes, subthemes, and representative quotes from interviews

Themes	Subthemes		Quotes
***Consult specific factors***	In person advantages	**Comfort factors** - In person visits provide more relief - Personal comfort	They just give us a little bit more relief because it’s one on one
		**Ease of describing symptoms**	Especially for me when you have physical symptoms and stuff like that is easier for doctors to kind of tell what is causing them instead over a camera
		**Encounters are more personal**	She's better one on one when she can actually see and touch the person as opposed to on the computer
	In person disadvantages	**Long wait times for appointments**	When you're at a … pediatric clinic … you sometimes can be waiting for quite some time
	Telemedicine advantages	**Staying close to home**	If I can have the answers on the comfort of my home and everything is working out so far for me, why not taking advantage of that
		**Telemedicine calls are just like talking in person**	It's like you're able to get the same information across. Like it's almost as if it is really face to face, but it's just through screen
		**Telemedicine is more than a phone call**	it's not just like a phone call so if he feels like something's off with her or she's not looking like herself he can even see that as well without actually having to be there
	Telemedicine disadvantages		
		**Technical hassles** - Awkward talking through the computer - Harder for clear communication - Some delay or disconnect in calls - Technical issues with telemedicine	It's a bit odd, because there is that little bit of a delay when you're talking to them from when they say something to when we actually kind of hear it
		**Telemedicine is a little rushed**	Sometimes it doesn’t last long and sometimes I have questions afterwards I should have ask him, but I couldn’t
***Context- specific factors***	In person advantages	**Likes a trip**	I like travelling to London because like we don't travel often, so I like to go there sometimes
	In person disadvantages	**Travelling is a Hassle** - In person visits are costly - Travelling wastes time	I cannot fly straight to London. I have to go to London and then go to Toronto and from Toronto to London.
	Telemedicine advantages	**Option relieves stress**	There's relief for me as a mom, because we do not have a specialist here in town, to have that contact
		**No dislikes about telemedicine**	I like, honestly, I like everything about it. I have nothing that I really dislike about it.
		**Telemedicine creates convenience** - Easy to travel to telemedicine station - Telemedicine is on time - Telemedicine saves time	Well, it’s usually after school, so I would just go right after school and it would only take max about an hour and it would be quick and it won’t really disrupt my day.
		**Telemedicine is cost-efficient**	You don't have to go through the process of booking a ticket or spending the money to get the ticket
		**Telemedicine is safe**	To me, it's way more comfortable to be here and it's safe because like I say, what is the difference between talking with you on the phone over me seeing you?
	Telemedicine disadvantages	**Frustration with telemedicine**	It's hard to talk to him because when you talk on the computer, you have to press the button to talk to him
		**Adolescent takes telemedicine less seriously – parent perception**	I think sometimes my daughter doesn't think it's as important, by doing the video
***Other factors mediating preference***	Adolescent condition	- Hospital in London is more efficient with tests - Less tests done for telemedicine - More tests can be done in person - Prefers tests done in the same hospital	I'll probably get blood work and ultrasound there [in London]. But if it's just like check-ups … we can do here [through telemedicine].
	**Testing factors**		
	Weather	**Preference depends on the weather**	If the weather is good I don’t sometimes mind going out to London … But in the winter or during worse weather I definitely prefer to stay in town and go for the appointment here
**Prefers telemedicine**			Well, there's almost no comparison it's so much easier doing the Tele-health appointment because it's not two airplanes and organizing our lives because we have two other children.
			It’s so much easier doing the telehealth appointment because it’s not two airplanes and organizing our lives because we have two other children. Personally, I prefer the telemedicine because just mainly for the reason that it takes hardly any time and I can get back to my everyday life.
**Prefers in-person**			Honestly, my daughter is so much happier when we're there [in person] and that it's despite the drive
			Person-to-person is still the much preferred option. I feel like it’s more personal scale. Like I think I understand it better like when I’m in the room with them.

### Consult-specific factors

#### In-person visits

During the interviews, participants identified consult-specific factors associated with preference for in-person consultations and telemedicine visits, categorized as advantages and disadvantages associated with both types of medical visit. Three consult-specific factors influencing preference for in-person consultations were expressed: comfort factors, ease of describing symptoms, and encounters being more personal.

Comfort factors innate to the in-person visits provided adolescents and caregivers more psychological relief seeing the physician in-person. Further, participants expressed that they took more comfort in communicating with the physician in-person. As one adolescent commented, “in-person visits are so much more normal and more comfortable” than telemedicine.

Participants also appreciated the ease of describing symptoms in-person and the ability to show the physician physical signs during their appointment. As one adolescent noted, “when you have physical symptoms and stuff like that is easier for doctors to kind of tell what is causing them instead over a camera.” During the interviews, participants discussed the comfort factor of encounters are more personal. “to see him in-person is a lot easier to talk to him in person.” This level of comfort seems to at least partially balance out the main stated disadvantage of long wait times. An adolescent explained that with in-person visits, “it takes a long time to wait and to see my doctor”.

#### Telemedicine visits

During the interviews, participants expressed several consult-specific advantages that telemedicine visits had over in-person consultations. Participants appreciated the ability to stay close to home. One adolescent explained, “it’s usually better when I’m at home.”.

Participants provided two related but differing perspectives on why telemedicine was favored over in-person consultations. Ten participants (7 adolescents and 3 caregivers) felt that there existed virtually no differences between a telemedicine visit and a phone or video chat. “During the visit it’s basically the same” one adolescent said. There were some participants who felt that during routine consultations, telemedicine offered the same advantages as in-person visits. “It’s like you’re able to get the same information across. Like it’s almost as if it is really face-to-face, but it’s just through a screen.”

Although some participants compared telemedicine to telephone calls some participants felt that telemedicine offered more than just a phone call. These participants felt that the face-to-face video interaction provided a more inclusive experience with the physicians. A caregiver explained that telemedicine was “a way to continue the relationship and the connection with the doctor.” Another patient explains the difference they see between telemedicine and in-person visits like this: “Compare it to like when I visit face-time my grandparents that live in Florida. They kind of see me but they don’t know how tall I am. It’s kind of different that way.” This quote acknowledges both the advantages and disadvantages of telemedicine visits.

The use of the conventional format of telemedicine (as used in the Ontario Telemedicine Network model) presents some challenges. Even though telemedicine created convenience, the pre-set duration of the telemedicine visit led to some adolescents and caregivers feeling that telemedicine visits are somewhat rushed and there may not be enough time to adequately discuss all issues.

One stated consult-specific disadvantage to telemedicine visits expressed by participants was the potential for adolescents to take telemedicine visits less seriously, as the physician was not physically present. This was a concern primarily expressed by caregivers, although not exclusively. One caregiver explained that when “the doctor is in your face, keeps the child more accountable too because they’re right there.”

### Context specific factors

#### In-person visits

There were few context-specific factors that participants stated as an advantage to in-person visits, other than participants saw an in-person visit as an opportunity to take a trip. As one adolescent said, “I like travelling to the center because like we don’t travel often, so I like to go there sometimes”.

This was a greater advantage for participants coming from further than 500 km away, although this advantage was often overshadowed by the disadvantages associated with the travel to in-person appointments, such as missing school, work and potential weather disruption.

Although in-person appointments were preferred for consult-specific reasons, caregivers and to a lesser extent, adolescents, felt that travelling for appointments was a hassle. In-person visits were considered costly and travelling an unproductive use of time.

#### Telemedicine visits

Participants expressed a number of advantages to telemedicine appointment. Almost all caregivers and adolescents felt that telemedicine was more convenient and efficient use of time and money. Almost all participants expressed dissatisfaction with the technical elements of telemedicine, such as lag during the consultation and disconnections during the visit. As a caregiver explains, “it’s a bit odd, because there is that little bit of a delay when you’re talking to them from when they say something to when we actually kind of hear it. You see that little bit of a pause going.” For some participants, these pauses made it easier to forget things, such as instructions from the doctor. This delay in communication as a disadvantage of telemedicine was expressed by almost all the participants. All participants including both adolescents and caregivers felt that telemedicine was a safe way to communicate with their physician.

### Factors mitigating preference

Factors mitigating the preference for type of visits were identified throughout the interviews. For routine and follow-up visits, participants preferred the telemedicine visits due to context-specific factors. Potentially inclement Canadian weather was one key factor in determining preference. One participant explained that telemedicine is “good for like a regular check-up.” Travelling for in-person appointments was seen by some adolescents as a nice trip during the summertime. However, in-person visits required adolescents and caregivers to miss time from work or school to travel for the appointment. When the weather was better, participants were more likely to favor in-person appointments over telemedicine visits.

Adolescent health condition and the reason for the visit also influenced expressed preference. If the appointment was a regularly scheduled routine appointment, telemedicine visits were preferred. However, if there was a change in patient condition or a specific concern to be addressed, in-person visits were preferred. When more extensive testing was required, the participants stated that they preferred to go to the hospital for an in-person visit. This was the most expressed factor related to preference for either type of visit.

## Discussion

This study assessed adolescent and caregiver attitudes towards virtual care and telemedicine in particular. We determined that preference for telemedicine for both adolescent and caregiver is dependent on consult-specific and context-specific factors, with adolescent diagnosis and weather conditions being the main modifying factors.

For dyads less than 500 km away from the center slightly more adolescents favored in person visits than their caregivers. We did not find a difference in the preference between telemedicine and tertiary care center if the adolescents lived more than 500 km away. This outcome might be partially due to younger adolescent’s underestimation of the amount of time it takes to travel to the tertiary care center. Furthermore, younger adolescents may experience more confounding reasons for preferring in-person visits such as being able to miss school or spending the day out with the caregivers.

Overall, there was acceptance of telemedicine, and factors contributing to attitudes between adolescents and caregivers were largely congruent. Factors driving preferences for in-person visits were consult-specific, and factors driving preferences for telemedicine were context specific. Even though our quantitative results show no significant relationship between the adolescent’s severity of illness and preference (sample size was small), our qualitative analysis shows that adolescent condition is still a strong mitigating factor contributing to preference of telemedicine vs. in-person visits. Adolescents with more complex conditions prefer in-person visits for a greater ease-of-mind. Conversely, adolescents with less complex conditions believed that telemedicine was comparable to in-person visits. As there were no strong dislikes for telemedicine, one adolescent expressed strong favoring for all in-person visits due to the ability for close in-person contacts with the physician. Therefore, it is important to respect pediatric adolescent’s attitudes when arranging telemedicine appointments to suit individual needs, especially for adolescents with more complex conditions. Most adolescents and caregivers agreed that having telemedicine as an option relieves stress and provides convenience. These findings are important because telemedicine is being utilized more often. Another major finding was related to concerns with telemedicine due to technical difficulties of the video calls. These challenges need to be addressed in the future.

Orlando and coworkers summarized patient and caregiver satisfaction with telemedicine information derived from 36 studies in adults [13]. The outcomes of satisfaction with telehealth were categorized into system experience, information sharing, consumer focus and overall satisfaction. There were high levels of satisfaction across all these dimensions. People living in rural and remote areas are generally satisfied with telehealth as a mode of service delivery as it may improve access to health care and avoid the inconvenience of travel [13].

Research on the pediatric use of telemedicine has been done predominantly in the setting of diabetes mellitus and is mostly focused on providers and telemedicine specialists [14]. Studies assessing attitudes towards telemedicine among caregivers have shown that caregivers are keen to pursue methods of virtual care because of convenience, cost and timeliness of information sharing [15]. Data on pediatric nephrology are scant. Peter Trnka published a descriptive retrospective study from Brisbane, Australia, with general acceptance and similar geographical challenges as our center without detailed interviews of the caregivers [16].

Less has been published with regards to the attitudes of adolescents. Stavas conducted semistructured interviews similar to us among 10 adolescents and 17 caregivers in the setting of child sexual abuse [17]. Stavas found an overwhelming positive response to telemedicine despite of the sensitive subject matter. While the adolescents felt scared, unprepared and nervous, they all expressed relief after the telemedicine visit [17]. We are unaware of additional studies that directly involved the adolescents.

Our study has a number of limitations. The adolescent group was heterogenous and may have suffered from selection bias. Because our qualitative study reached saturation of themes so early in the study, there were insufficient numbers in order for us to attempt to differentiate experience with the health services and attitudes with telemedicine. Additionally, as the interviews were conducted over the phone, there might be slight discrepancies between the transcribed version of the phone calls and what the participants intended to say. Suboptimal phone connection, loud background noise, and heavy accents from patients can all lead to potential misunderstanding. To minimize the discrepancy, transcription was first performed through NoNotes, and further reviewed manually by the researchers. Due to the age difference in the adolescents, some adolescents were also better at communicating their thoughts than others. In times when adolescents were not able to provide clear answers, the interviewer had to improvise and guide the conversation further, which may lead to bias and confounding results. However, bias is minimized as interviewers were provided with a standard set of interview questions to follow. Finally, as next steps it would be interesting to perform analysis within each caregiver-adolescent dyad and investigate how their relationship can have an impact on preference. Nonetheless, the data presented here demonstrate the complex aspects that affect the attitude towards telemedicine and highlights that the adolescents may have different attitudes towards telemedicine when compared to their caregivers. Our study concluded prior to the COVID-19 pandemic. Future research should include the reassessment during the COVID-19 pandemic, expand on platforms for the conduct of virtual care, and address the technical difficulties which have been highlighted in our findings.

## Conclusions

Indiscriminate transfer to chronic care predicated on mainly telemedicine approach is not compatible with user expressed attitudes (especially adolescents). Understanding the preferences and perceptions of telemedicine, by users, is essential for best utilization of virtual care of the adolescent in the out-patient setting. Appreciation of the experiences and preferences of adolescents, relative to caregivers, will allow intelligent design and delivery of telemedical consultation in a way most appropriate to the kind of consultation. This study also shows that we need to encourage our youth to speak up about their preferences and respect that they may prefer in person visits over virtual encounters.

## Supplementary Information

**Additional file 1.**

## Data Availability

The datasets used and/or analyzed during the current study are available from the corresponding author on reasonable request.
