# Supplementary material for: Adolescent and caregiver attitudes towards telemedicine use in pediatric nephrology
Source: BMC Health Serv Res. 2021 Jun 1;21:537. doi: 10.1186/s12913-021-06506-0 (PMC8169397; doi:10.1186/s12913-021-06506-0)
Supplement: Supplementary file 1 — Additional file 1. [file 12913_2021_6506_MOESM1_ESM.docx]

Appendix A:

Questions for the parent/caregiver of paediatric nephrology patients living in remote locations receiving telemedicine services rather than in-person clinic visits for prescreening.

1. What is your postal code (to calculate the distance to the nearest paediatric nephrology centre)?
2. Is that a village (<1000), a town or a city (>50,000)?
3. What is your highest degree (some high school, high school, one post-secondary degree, more than one post-secondary degree)?
4. Which paediatric nephrology centre to you to to?
5. How long does it take to get there?
6. Do you need accommodation for a clinic vist?
7. How many meals do you typically encounter during a clinic visit in the nearest paediatric nephrology centre?
8. Can you drive there or do you need to fly?
9. If flying, how many flight legs?
10. Where do you go for telemedicine?
11. What is the address of the telemedicine station (include postal code to calculate the distance to the nearest telemedicine station)?
12. How do you get there?
13. Why do you see a paediatric nephrologist?
14. When did you first see your paediatric nephrologist?
15. How many times have you seen your paediatric nephrologist in person?
16. How many times have you seen your paediatric nephrologist on telemedicine?
17. How long does it take you to travel to the clinic in London?
18. How long does it take you to travel to the telemedicine station?
